# Are third-generation cephalosporins unavoidable for empirical therapy of community-acquired pneumonia in adult patients who require ICU admission? A retrospective study

**DOI:** 10.1186/s13613-017-0259-4

**Published:** 2017-03-24

**Authors:** Geoffroy Hariri, Jacques Tankovic, Pierre-Yves Boëlle, Vincent Dubée, Guillaume Leblanc, Claire Pichereau, Simon Bourcier, Naike Bigé, Jean-Luc Baudel, Arnaud Galbois, Hafid Ait-Oufella, Eric Maury

**Affiliations:** 1 Service de Réanimation Médicale, Hôpital Saint-Antoine, Assistance Publique-Hôpitaux de Paris, 184 rue du faubourg Saint-Antoine, 75571 Paris, France; 2 Microbiologie, Hôpital Saint-Antoine, Assistance Publique-Hôpitaux de Paris, Paris, France; 3 Institut Pierre-Louis d’Epidémiologie et de Santé Publique, U 1136, Inserm, 75012 Paris, France; 4 UPMC Univ Paris 06, Sorbonne Universités, Paris, France; 5 Réanimation Polyvalente, HP Claude Galien, 91480 Quincy-sous-Sénart, France

**Keywords:** Community-acquired pneumonia, Third-generation cephalosporins, Extended-spectrum beta-lactamases, Intensive care unit, Antibiotics

## Abstract

**Background:**

Third-generation cephalosporins (3GCs) are recommended for empirical antibiotic therapy of community-acquired pneumonia (CAP) in patients requiring ICU admission. However, their extensive use could promote the emergence of extended-spectrum beta-lactamases-producing *Enterobacteriaceae*. Our aim was to assess whether the use of 3GCs in patients with CAP requiring ICU admission was justified.

**Methods:**

We assessed all patients with CAP who required ICU admission during a 7-year period. We recorded empirical and definitive antibiotic therapies and susceptibility of causative pathogens. Amoxicillin, amoxicillin/clavulanate (A/C) susceptibilities as well as amikacin susceptibility of A/C-resistant strains were recorded.

**Results:**

From January 2007 to March 2014, 391 patients were included in the study. Empirical 3GCs were used in 215 patients (55%). Among 267 patients with microbiologically documented CAP (68%), 241 received a beta-lactam as definitive therapy, and of those, 3CGs were chosen for 43 patients (18%). Amoxicillin or A/C was active against isolated pathogens in 159 patients (66%), while 39 patients (16%) required a beta-lactam with a broader spectrum than 3GCs. Ninety-four per cent of A/C-resistant strains were amikacin susceptible.

**Conclusions:**

In ICU patients with CAP, 3GCs given on an empirical basis are changed, according to microbiological documentation, for another beta-lactam in 82% of cases especially to A/C in the absence of resistance risk factor. In patients evidencing risk factors for A/C-resistant strains infection, 3GCs or antipseudomonal beta-lactams including carbapenem associated with amikacin in the most severe patients seem a relevant empirical antibiotic therapy. This strategy could decrease 3GCs’ use.

## Background

Community-acquired pneumonia (CAP) is a major cause of hospitalization and death worldwide [1, 2]. CAP is the leading infectious cause for admission in the intensive care unit (ICU) and one of the most common causes of sepsis, severe sepsis and septic shock [3, 4]. Guidelines published in 2006 by the French Intensive Care Society (SRLF) and the French Infectious Diseases Society (SPILF) [5] recommended a non-antipseudomonal third-generation cephalosporin (3GC, as ceftriaxone or cefotaxime) in combination with either a macrolide or a fluoroquinolone for empirical treatment of patients with severe CAP who require ICU admission. Since the publication of those recommendations, a sharp increase in 3GCs prescription has been observed, with a 58% increase in ceftriaxone use in France between 2008 and 2013 (*French National Institute for Public Health Surveillance, RAISIN*) [6]. Between 2003 and 2012, the incidence of third-generation cephalosporin-resistant strains, especially extended-spectrum beta-lactamases-producing *Enterobacteriaceae* (ESBLEs), increased from 0.17 to 0.48 per 1000 hospitalization days [7].

Furthermore, the increased incidence of ESBLEs promotes the use of carbapenems, which in turn could increase the risk of emergence and spreading of carbapenem-resistant strains. A restricted use of 3GCs could therefore be warranted [9], as it might provide a possible strategy to decrease incidence of 3GCs’ resistance [10]. Thus, the aim of our study was to assess whether the use of 3GCs in patients with CAP who require ICU admission was justified based on the microbiologically identified causative pathogen and its susceptibility pattern.

## Methods

The study was conducted in a 670-bed tertiary teaching hospital in Paris, France. We retrospectively reviewed medical records of all consecutive adult patients who required ICU admission between January 2007 and March 2014 for acute respiratory failure. Pneumonia was diagnosed with the following criteria: a new alveolar, interstitial or alveolo-interstitial opacity on chest radiography, associated with at least two of the following: cough, sputum production, temperature above 38 °C or below 35 °C, and auscultatory findings consistent with pneumonia or dyspnoea. To be considered a community-acquired pneumonia, symptoms had to develop in the community or within the first 48 h after hospital admission [11–13]. Patients were admitted to the ICU from the emergency department or directly from the pre-hospital emergency medical team. The institutional review board of our institution approved the study.

For each patient included in the study, the following parameters were recorded: age, sex, underlying comorbidities (chronic lung disease, hypertension, diabetes, chronic liver disease, heart failure, chronic renal disease, HIV infection, ongoing solid cancer or blood cancer), SAPS II score, mechanical ventilation use, intravenous vasopressors administered for septic shock and ICU mortality.

A pathogen was considered causal for CAP after a comprehensive and critical appraisal of clinical and microbiological data. The microbiological diagnostic workup for all patients included: blood cultures obtained upon admission and before antibiotic administration, expectorated sputum sample in patients able to cough or blind endotracheal aspirations in patients mechanically ventilated. A broncho-alveolar lavage through fibroscopy was performed if others means were deemed unsuccessful. Respiratory samples were analysed for Gram staining initially and after 48 h of culture. According to international guidelines, immunochromatographic tests for detection of *Streptococcus pneumoniae* and *Legionella pneumophila* serogroup 1 antigen in urine were also performed [5]. The addition of PCR virus detection on lower respiratory samples or nasal–pharyngeal swabs (e.g. specific influenza PCR testing during the flu-season or respiratory virus PCR panel), or direct detection and quantitative PCR assessment of *Pneumocystis jirovecii* on broncho-alveolar fluid in patients with underlying immunosuppression was left at the discretion of the attending intensivist.

As per French guidelines, at our institution the combination of ceftriaxone or cefotaxime with either a macrolide or a fluoroquinolone is used as empirical treatment of patients with severe CAP requiring ICU admission. Then, as suggested by internal protocols, empiric antimicrobial therapy would be reassessed after 48 h by attending physicians and adjusted according to the microbiological cultures, if available. The most narrow-spectrum antibiotic would be used as the definitive therapy based on antimicrobial susceptibility results based on the antibiogram diameter inhibition and switched to the oral route as soon as possible. If pneumococcal pneumonia were documented, the association of a beta-lactam and a macrolide would be maintained for the first 3 days [14] and then a beta-lactam (usually amoxicillin) would be maintained alone thereafter. If all cultures would remain negative in a patient having received any antibiotic before microbiological samples, the empiric therapy would be switched to oral pristinamycin. However, special attention would be paid to the direct examination of the sputum specimen in those patients, as the presence of typical Gram stain aspects (e.g. Gram-positive diplococci or numerous Gram-negative bacilli) might modify the choice of definitive therapy. In general, fluoroquinolones use was discouraged except in case of confirmed severe legionellosis [15].

The appropriateness of the 3GCs utilization was assessed in patients with microbiologically documented pneumonia, based on the susceptibility of the identified pathogens. Since Infectious Diseases Society of America/American Thoracic Society Consensus Guidelines recommend 3GC or combination of amoxicillin/β-lactamase inhibitor in case of severe CAP [11], special attention was paid to amoxicillin/clavulanate (A/C) susceptibility. The empirical use of 3GCs was therefore classified as:

- Unjustified when the pathogen was susceptible to amoxicillin or amoxicillin/clavulanate (A/C);
- Appropriate when the pathogen was resistant to amoxicillin/clavulanic acid and susceptible to non-antipseudomonal 3GCs;
- Insufficient when the pathogen was resistant to both amoxicillin/clavulanate and non-antipseudomonal 3GCs and required a broader-spectrum beta-lactam.

In patients presenting with CAP exhibiting A/C-resistant strains, we revised the charts to explore the presence of underlying comorbidities previously reported as risk factors for resistant pathogens [16] (e.g. chronic lung disease, hypertension, diabetes, chronic liver disease, heart failure, renal failure, HIV infection, stroke, ambulatory status, previous antibiotic treatment and progressive cancer). In those patients, we assessed the susceptibility to aminoglycosides (amikacin and gentamicin).

### Statistical analysis

Data are presented as numbers and percentages for categorical variables and mean ± standard deviation (SD) for continuous variables. Comparison between groups was made using Pearson’s Chi-squared test. *P* values <0.05 indicated statistical significance. Odds ratios for risk factors for A/C resistance were determined by logistic regression. An univariable analysis was first performed, and variables showing association with A/C resistance (*P* < 0.15) were included in a multivariate analysis, to calculate odds ratios. All analyses were performed using R 2.11.1 statistical package.

## Results

**Fig. 1:**
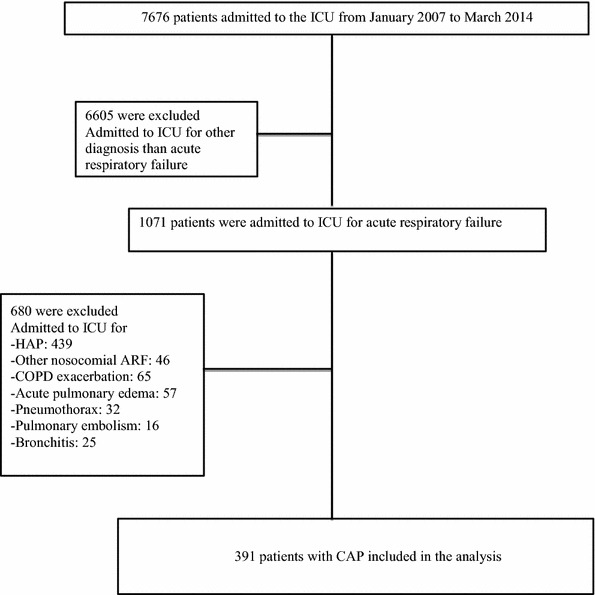
Flow chart of patients included in the analysis. *HAP* hospital-associated pneumonia, *ARF* acute respiratory failure, *COPD* chronic obstructive pulmonary disease

Between January 2007 and March 2014, 7676 patients were admitted in the ICU. Out of 1071 patients hospitalized in the ICU for acute respiratory failure, a final diagnosis of CAP was made in 391 patients (Fig. 1).

Of these 391 patients (55% male, mean age: 65 ± 19 years, SAPS II score of 47 ± 22), 172 (44%) required mechanical ventilation and 107 (27%) received vasopressors. Overall mortality during the ICU stay was 18% (73 patients). Patients’ characteristics are presented in Table 1.

**Table 1:** Patients’ characteristics

Characteristics	*N*	%
Sex (male)	215	55.0
Age	65 ± 19	
IGSII	47 ± 22	
Chronic lung disease	119	30.4
HTA	142	36.3
Diabetes mellitus	57	14.6
Congestive heart failure	43	11.0
Chronic liver disease	41	10.4
Chronic renal disease	23	5.9
Stroke	19	4.9
Progressive cancer	22	5.6
Blood cancer	37	9.5
HIV	20	5.1
Other causes of immunosuppression	8	2.0
Mechanical ventilation	172	44.0
Catecholamines	107	27.3
Acute renal failure	16	4.1
Renal replacement therapy	1	0.3
Mortality	73	18.6

The initial empiric antibiotic therapy consisted in a monotherapy in 53 patients (13%), a bitherapy in 312 patients (80%) and a tritherapy in 21 patients (5%). The bitherapies consisted in beta-lactam and macrolide (*n* = 277), beta-lactam and metronidazole (*n* = 34) and sulphonamides and macrolide (*n* = 1). The beta-lactams more frequently used were: a non-antipseudomonal 3GC in 215 patients (55%) and amoxicillin/clavulanate in 75 patients (19%) (Table 2). Macrolides were empirically prescribed in 277 patients (70%). Oseltamivir was associated with antibiotic therapy on an empirical basis in 23 patients.

**Table 2:** Anti-infective therapies administered on an empirical basis to the 391 patients with community-acquired pneumonia

Empirical antibiotic therapies	*N* (%)
Macrolides	277 (71)
Third-generation cephalosporin	215 (55)
Amoxicillin/clavulanate	75 (19)
Piperacillin/tazobactam	64 (16)
Metronidazole	34 (12)
Oseltamivir	23 (6)
Cotrimoxazole	18 (5)
Fluoroquinolone	15 (4)
Aminoglycoside	9 (2)
Imipenem	9 (2)
Amoxicillin	7 (2)
Vancomycin	6 (2)
Ceftazidime	3 (1)

### Microbiologically proven community-acquired pneumonia

A causative pathogen was identified in 267 patients (68%) (Table 3). *Streptococcus pneumoniae* and *Haemophilus influenzae* were the most frequent pathogens, identified in 43.1 and 17.6% of cases, respectively.

**Table 3:** Distribution of pathogens identified in 267 patients with documented community-acquired pneumonia

Microbiological strains	*n* (%)
*Streptococcus pneumonia*	115 (43.1)
*Haemophilus influenza*	47 (17.6)
*Moraxella catarrhalis*	4 (1.5)
*Legionella pneumophila*	7 (2.6)
*Mycoplasma pneumoniae*	1 (0.3)
Enterobacteriaceae	
*Escherichia coli*	22 (8.2)
*Klebsiella pneumoniae*	15 (5.6)
*Klebsiella oxytoca*	3 (1.1)
*Serratia marcescens*	4 (1.5)
*Enterobacter cloacae*	4 (1.5)
*Morganella morganii*	3 (1.1)
*Proteus mirabilis*	2 (0.7)
*Hafnia alvei*	1 (0.3)
*Enterobacter aerogenes*	1 (0.3)
Non-fermenting Gram-negative bacteria	
*Pseudomonas aeruginosa*	22 (8.2)
*Stenotrophomonas maltophilia*	2 (0.7)
*Acinetobacter baumannii*	1 (0.3)
Methi-S *Staphylococcus aureus*	18 (5.7)
*Methi*-*R Staphyloccocus aureus*	3 (1.1)
*Corynebacterium*	3 (1.1)
*Fusobacterium other*	1 (0.3)
*Neisseiria* sp. other	1 (0.3)
*Streptococcus anginosus other*	1 (0.3)
*Streptococcus milleri other*	2 (0.3)
*Pneumocystis jirovecii*	11 (4.1)
Viruses	
H1N1 influenzae	4 (1.5)
Adenovirus	1 (0.3)
*Mycobacterium tuberculosis*	1 (0.3)

The total count of pathogens exceeds the total number of documented pneumonia since 33 pneumonias were caused by two pathogens

**Fig. 2:**
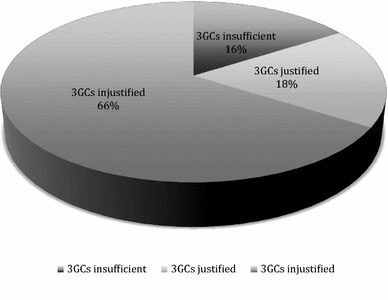
Relevance of third-generation cephalosporins given on an empirical basis to patients with documented community-acquired pneumonia and requiring at least a beta-lactam

Among the 267 patients with microbiologically proven CAP, 241 were given a beta-lactam alone as a definitive therapy (Table 4). In these patients, the empirical prescription of 3GCs was unjustified in 159 patients (66%) and was changed for amoxicillin/clavulanate (*n* = 70), amoxicillin (*n* = 87) or oxacillin (*n* = 2). The empirical 3GC’s prescription was appropriate and continued in 43 patients (18%) and insufficient in 39 patients (16%) in whom a broader-spectrum beta-lactam was required for definitive therapy. Finally, in patients with documented pneumonia requiring beta-lactam, non-antipseudomonal 3GCs prescribed on an empirical basis were justified in less than a fifth of patients (Fig. 2).

**Table 4:** Empiric and definitive beta-lactams given to 241 patients with documented pneumonia requiring at least a beta-lactam

Beta-lactam	*n* (%)
Non-antipseudomonal 3GC unjustified	159 (65.9)
Amoxicillin	87 (36.1)
Amoxicillin/clavulanate	70 (29)
Oxacillin	2 (0.8)
Non-antipseudomonal 3GC appropriate	43 (17.8)
Non-antipseudomonal 3GC inappropriate	39 (16.2)
Piperacillin/tazobactam	16 (6.7)
Ticarcillin	9 (3.7)
Carbapenem	7 (2.9)
Ceftazidime	3 (1.2)
Tazocillin/clavulanate	2 (0.8)
Cefepime	1 (0.4)
Piperacillin	1 (0.4)

No pneumococcal strain exhibited resistance to amoxicillin. Among *H influenza* strains, 43% were fully amoxicillin susceptible and 36% were amoxicillin resistant and A/C susceptible, whereas 21% were A/C resistant and cefotaxime susceptible. All *Haemophilus* strains were susceptible to spiramycin. Resistance to cefotaxime was mainly observed in non-fermenting Gram-negative bacilli. All patients with *Pseudomonas aeruginosa-*associated CAP had at least one of the specific risk factor previously defined in French guidelines [17].

Resistance to amoxicillin/clavulanate was observed in 82 patients and was more frequent among patients with comorbidities (Table 5). In the multivariable analysis, variables significantly associated with resistance to amoxicillin/clavulanate were chronic lung disease [OR 1.9 (95% CI 1–3.6)], cancer [OR 4.5 (95% CI 2.0–9.8)], previous antibiotic therapy [OR 2.7 (95% CI 1.3–5.9)], limited autonomy [OR 2.9 (95% CI 1.0–8.2) and stroke [OR 0.1 (95% CI 0.0–0.9)].

**Table 5:** Univariable and multivariable analysis of comorbidities associated with infection due to amoxicillin/clavulanate non-susceptible

Comorbidities	Univariable analysis		Multivariable analysis	
	OR (95% CI)	*P* value	OR (CI 95%)	*P* value
Arterial hypertension	1.3 (0.7–2.2)	0.40		
Chronic lung disease	1.7 (0.9–3.1)	0.09	1.9 (1.0–3.6)	0.05
Chronic renal failure	1.6 (0.4–6.0)	0.52		
Chronic liver disease	0.7 (0.3–1.6)	0.36		
Congestive heart failure	1.2 (0.4–3.3)	0.78		
Immunosuppression (HIV, other)	2.0 (0.8–5.1)	0.13		
Diabetes mellitus	1.0 (0.4–2.4)	0.97		
Cancer	4.3 (2.1–9.1)	0.001	4.5 (2.0–9.8)	0.04
Stroke	0.2 (0.0–1.2)	0.07	0.1 (0.0–0.9)	0.03
Nursing home/non-ambulatory^b^	2.1 (0.9–5.0)	0.11	2.9 (1.0–8.2)	0.04
Proton pump inhibitor	1.1 (0.5–2.1)	0.85		
Other immunosuppression	2.4 (0.7–8.1)	0.16		
Hospitalization^a^	1.6 (0.8–3.4)	0.23		
Antibiotic during last 90 days	2.7 (1.3–5.6)	0.01	2.7 (1.3–5.9)	0.01

^a^At least 2 days in the last 90 days

^b^Non-ambulatory status was defined as being bedridden or using a wheelchair because of difficulty walking

Among the 82 pathogens resistant to amoxicillin/clavulanate, 18 strains (22%) were resistant to gentamicin, whereas five strains (6%) were resistant to both gentamicin and amikacin.

## Discussion

In our retrospective study, we evaluated the efficacy and relevance of the chosen empirical antibiotic therapy especially non-antipseudomonal 3GCs in the light of the microbiologically identified pathogens and their susceptibility result. To assess whether 3GCs were really justified, we focused on CAP with microbiological documentation, which required definitive beta-lactam therapy. These infections were separated into three groups according to whether 3GCs appeared unjustified (pathogen susceptible either to amoxicillin or amoxicillin/clavulanate), appropriate (pathogen cefotaxime susceptible and resistant to amoxicillin/clavulanate) or insufficient (pathogen resistant to both amoxicillin/clavulanate and non-antipseudomonal 3GCs and requiring a broader-spectrum beta-lactam).

In the present study, 3GCs were the second most frequent empirical antibiotic used for severe CAP after macrolides (53 and 70%, respectively). This is in agreement with French guidelines [5]. *Streptococcus pneumoniae* and *Haemophilus influenzae* remain, as previously observed [18–22], the most frequent bacterial species causing CAP. These two pathogens accounted for 60% cases of documented pneumonias. These pathogens are most often highly susceptible to narrow-spectrum β-lactams such as amoxicillin or amoxicillin/clavulanate [20], except for some *H. influenzae* isolates. In the present studies, 10 *H influenzae* strains (21%) were amoxicillin/clavulanate resistant, but all were susceptible to both cefotaxime and spiramycin.

The group of unjustified third-generation cephalosporin prescription is the most important with 66% (159) of patients with documented infection; in this group, treatment with amoxicillin or amoxicillin/clavulanate would have been sufficient. Appropriate 3GC’s prescription was observed in only 18% (43 patients) of our cohort. Finally, the group in which third-generation cephalosporin use appeared insufficient represents 16% (39) of patients, meaning that in these patients a beta-lactam with a broader spectrum than 3GCs would have been required.

These results suggest that the use of third-generation cephalosporins as an empiric beta-lactam for treatment of severe community-acquired pneumonia admitted to the ICU is a perfectly tailored antibiotic therapy in only one out of five patients. Most of the time, a narrower-spectrum beta-lactam might be preferred. The amoxicillin/clavulanate combination would be a satisfactory alternative to 3GCs against two-thirds of overall recovered pathogens. Moreover, non-antipseudomonal 3GC spectrum appears insufficient in one out of six patients with CAP requiring beta-lactam.

In a second part of the study, we sought to delineate patients for whom amoxicillin/beta-lactamase inhibitor would be insufficient and who would require a broader-spectrum beta-lactam. For that, we assessed the presence or absence of risk factors associated with colonization/infection with resistant strains previously reported [21]. We found that chronic lung disease, ongoing cancer, non-ambulatory status and previous antibiotic therapy were significantly associated with amoxicillin/clavulanate-resistant strains.

These amoxicillin/clavulanate-resistant isolates (mainly *Pseudomonas aeruginosa*, non-fermenting Gram-negative bacilli and several *Enterobacteriaceae*) were most of the time sensitive to amikacin (94%), suggesting that this antibiotic could be considered in patients with risk factors for infection with an amoxicillin/clavulanate non-susceptible pathogen.

Prior use of 3CGs has been identified as a risk factor for infections caused by ESBL-producing *Enterobacteriaceae* [8, 23, 24]. Recent experiments report a decrease in ESBLEs emergence following decrease in cephalosporins use [10]. These data support, when possible, a decrease in the use of 3CGs to control ESBL-producing *Enterobacteriaceae* spreading. According to our results, amoxicillin/clavulanate could be proposed as an alternative beta-lactam for severe CAP in patients without risk factors for amoxicillin/clavulanate resistance. This situation (absence of A/C resistance risk factor) is observed in 55% of patients.

Such a strategy could contribute to limit the 3GCs use and then probably the emergence of extended-spectrum beta-lactamases (ESBLs). It is important to note that conversely to French recommendations, amoxicillin/beta-lactamase inhibitor is proposed as a possible first-line empirical beta-lactam for ICU patients with CAP in North American [11] and British [25] guidelines. Reducing the use of third-generation cephalosporins for CAP is already suggested in the emergency department [26].

Among patients with risk factors for amoxicillin/clavulanate resistance, strains encountered are very similar to those observed in patients with healthcare-associated pneumonia, and even this is still debated, to strains identified in patients with ventilator-associated pneumonia and/or hospital-acquired pneumonia [16–27]. The latter patients should receive at least 3GCs for empirical treatment and antipseudomonal beta-lactam (piperacillin/tazobactam, cefepime, ceftazidime) or carbapenem associated with an aminoglycoside for the most severe patients. Amikacin appears in the present study more frequently efficient than gentamicin.

The present study has, however, several limitations. First, whereas some of the observed data are similar to previously published results, the present study is a single-centre retrospective study with a moderate size population. Thus, these results should be interpreted cautiously and confirmed in a larger population. Second, our approach relies on an extensive infectious workup including invasive procedures, which is a strategy not supported by current guidelines in non-immunocompromised patients. Nevertheless, owing to significant increase in antibiotic resistance and to the necessity of a relevant antimicrobial stewardship, an aggressive diagnostic approach dedicated to increase the rate of microbiological identification in order to use the antibiotic therapy with the narrowest spectrum seems mandatory.

## Conclusion

In ICU patients with documented community-acquired pneumonia who require definitively a beta-lactam, we observed that 3GCs are most of the time an unjustified or insufficient antibiotic therapy. Based on the present retrospective analysis, a narrower-spectrum beta-lactam antibiotic with amoxicillin/clavulanate combined with a macrolide could replace 3GCs as empirical antibiotics for severe CAP in patients without risk factors for amoxicillin/clavulanate resistance, i.e. chronic lung disease, ongoing cancer, non-ambulatory status and antibiotic therapy during the previous 90 days. In patients with risk factors for amoxicillin/clavulanate resistance, 3GCs, antipseudomonal beta-lactam including carbapenem associated with amikacin in the most severe patients seems a relevant empirical antibiotic therapy. This strategy could promote a decrease in 3GCs’ consumption.

## Abbreviations

3GC: third-generation cephalosporin
ESBL: extended-spectrum beta-lactamase
ICU: intensive care unit
CAP: community-acquired pneumonia
HCAP: healthcare-associated pneumonia
A/C: amoxicillin/clavulanate
